# Phylogenetic distribution and predominant genotype of the avian infectious bronchitis virus in China during 2008-2009

**DOI:** 10.1186/1743-422X-8-184

**Published:** 2011-04-22

**Authors:** Jun Ji, Jingwei Xie, Feng Chen, Dingming Shu, Kejing Zuo, Chunyi Xue, Jianping Qin, Hongmei Li, Yingzuo Bi, Jingyun Ma, Qingmei Xie

**Affiliations:** 1 College of Animal Science, South China Agricultural University, Guangzhou 510642, China; 2 Guangdong Wen's Foodstuffs Group Co. Ltd., Yunfu 527439, China; 3 State Key Laboratory of Livestock and Poultry Breeding, Institute of Animal Science, Guangdong Academy of Agricultural Sciences, Guangzhou 510640, China; 4 State Key Laboratory of Biocontrol, College of Life Sciences, Sun Yat-Sen University, Guangzhou 510006, China

## Abstract

**Background:**

The nephropathogenic avian infectious bronchitis (IB) caused unprecedented economic losses to the commercial chicken industry of China in 2008-2009. To investigate the prevalence of nephropathogenic IB in China, eighty IBV isolates from different provinces during 2008-2009 were identified by dwarf embryo test and RT-PCR.

**Results:**

The strains were mostly isolated in winter and spring with a wide age range of IB outbreaks, from 4 to 69 days. By the virus recovery trials, 70/80 of the strains resulted in the deaths or distresses of birds from nephritis. To learn more about the molecular evolutionary characteristics of the circulating field strains, the coding region of major spike 1 (S1) protein gene of these strains was RT-PCR amplified and sequenced. Compared to the published representative strains, nucleotides and amino acids sequence analysis indicated that the S1 genes of these strains and the reference strains displayed homologies ranging from 75.1% to 99.8% and from 73.1% to 99.8% respectively. S1 protein of the major pandemic strains contained 540 or 542 amino acids with the cleavage site of HRRRR or RRFRR. Phylogenetic analysis revealed that recent field isolates of IBV in China were mostly belonged to A2-branch (QXIBV-branch) and HN08-branch, only one isolate was belonged to Gray-branch and M41-branch respectively. Most of the 80 strains showed evolutionarily distant from vaccine strains.

**Conclusions:**

The results of this study suggested that nephropathogenic IBVs were mainly A2-like strains in China during 2008-2009.

## Background

Infectious bronchitis (IB) is a serious and highly contagious disease of chickens, accompanied by decreased egg production and poor egg quality in laying flocks. Avian infectious bronchitis virus (IBV) was first reported in the USA, replicating in the respiratory tract and some epithelial cells of gut, kidney, and oviduct [1-3]. IBV commonly predisposed the birds to secondary infection with some bacterium, such as *Escherichia coli *and *Mycoplasma gallisepticum*, resulting in complicated disease process and increased mortality [4,5]. The clinical disease and production problems frequently cause catastrophic economic losses to the poultry industry all over the world. IBV belongs to the genus *Coronaviridae*, family *Coronaviridae*, order *Nidovirales*, and possesses a single stranded positive-sense RNA genome encoding four structure proteins, phosphorylated nucleocapsid (N) protein, small envelope protein (E), integral membrane glycoprotein (M), and spike glycoprotein (S) [6,7]. The S glycoprotein on the outside of the virus contains epitopes associated with serotype differences, and is cleaved post-translationally by cellular proteases into the S1 and S2 subunits [8,9]. The globular S1 subunit forms the tip of a spike, extending outward, plays a role in attachment and entry into the host cell, which has relation to induce virus neutralizing antibody and hemagglutination inhibition antibody, whereas the S2 subunit anchors the S1 moiety to the viral membrane [8-11]. Coding for the heavily glycosylated spike glycoprotein, the error-prone nature of RNA polymerase made the S1 gene could easily generate nucleotide insertions, deletions, point mutations, and RNA recombination under vaccine pressure, to bring about new variation strain and change of tissue tropism [12-16]. It is documented that only a few amino acid differences amongst S proteins are sufficient to have a detrimental impact on cross-protection [15,17-20]. Antigenically different serotypes and newly emerged variants of field chicken flocks lead to vaccine breaks [21,22].

Recently, more than 20 serotypes within IBV have been identified worldwide. The complex epidemiology characterize of IB raised the control difficulty. In China, since IBV strains were first isolated and identified in 1982, various live-attenuated and inactivated vaccines derived from Massachusetts (Mass) serotype strains have been widely and extensively used in chicken farms to reduce the adverse effect of the IBV [23,24]. However, the disease continues to emerge and cause serious production problems, even occurred in routinely vaccinated layer and breeder flocks in China, and the situation gets worse as time progressed [25].

It was documented that nephropathogenic type IB has become more and more prevalent in China. The unprecedented economic losses caused by the nephropathogenic IB suggested that selecting the appropriate vaccine strain against the IB outbreaks is of great importance [25,26]. However, the integrated natures of novel circulating IBV strains in mainland China were not well-learned.

The previous study by other researchers has been revealed that the variation in S1 sequences was closely confirmed relative to the emergence of novel strains, and S1 gene sequence was a good predictor of challenge of immunity in chickens [17,18,27]. This study was conducted to identify the IBV strains that have escaped immune defenses conferred by vaccination in China. The genetic characterization of recent IBV field isolates in China was performed by sequencing the whole S1 genes, sequence alignment and phylogenetic analysis compared with other reference strains.

## Results

### Eighty IBV strains isolated during 2008-2009 in China

From unhealthy birds suspected of IBV infection in the vaccinated chicken flocks from Guangdong, Guangxi, Fujian, Hainan, Jiangsu, Zhejiang, Chongqing, Hubei, Sichuan and Jiangxi province of China, 80 filed IBV strains were isolated during 2008-2009. The isolation rates in the two years were season-dependent to some extent, 30 strains were isolated in October, while only seven strains were isolated in summer (from June to August). The ages of flocks at the time of the outbreak varied between 4 and 69 days. Most of the strains were isolated from the chickens between 10 to 30 days of age. The detailed clinical record of each strain was showed in Table 1.

**Table 1:** IBV strains isolated from flocks in different provinces of China

Virus Strain	Abbreviation	Major clinical signs	Age of IB outbreak (days)	Accession number
CK/CH/Chongqing/0908	CQ8	Respiratory	16	GU938413
CK/CH/Chongqing/0909	CQ9	Nephritis	38	GU938414
CK/CH/Sichuan/Meishan/0910	MS1	Nephritis	57	GU938415
CK/CH/Fujian/Putian1/0910	PT1	Nephritis	20	GU938408
CK/CH/Fujian/Putian2/0910	PT2	Nephritis	16	GU938409
CK/CH/Fujian/Putian3/0910	PT3	Nephritis	19	GU938410
CK/CH/Fujian/Putian4/0910	PT4	Nephritis	19	GU938411
CK/CH/Guangdong/Baitu/0904	BT	Nephritis	32	GU938385
CK/CH/Guangdong/DashaY4/0902	Y4	Nephritis	23	GU938368
CK/CH/Guangdong/DashaY9/0902	Y9	Nephritis	23	GU938367
CK/CH/Guangdong/Fengmulang/0901	FML	Nephritis	20	GU938441
CK/CH/Guangdong/Heyuan/0902	HY	Nephritis	27	GU938443
CK/CH/Guangdong/Heyuan1/0904	HY41	Nephritis	25	GU938386
CK/CH/Guangdong/Heyuan2/0904	HY42	Nephritis	18	GU938387
CK/CH/Guangdong/Heyuan1/0905	HY51	Nephritis	34	GU938401
CK/CH/Guangdong/Heyuan2/0905	HY52	Nephritis	55	GU938416
CK/CH/Guangdong/Heyuan1/0910	HY1	Nephritis	15	GU938406
CK/CH/Guangdong/Heyuan2/0910	HY2	Nephritis	32	GU938407
CK/CH/Guangdong/Heyuan3/0910	HY3	Nephritis	60	GU938421
CK/CH/Guangdong/Keyanjidi/0908	KY	Nephritis	23	GU938402
CK/CH/Guangdong/Lezhu1/0905	LZ1	Nephritis	4	GU938397
CK/CH/Guangdong/Lezhu2/0905	LZ2	Respiratory	13	GU938398
CK/CH/Guangdong/Lezhu3/0905	LZ3	Nephritis	13	GU938388
CK/CH/Guangdong/Lianhua/0806	LH	Respiratory	15	GU938366
CK/CH/Guangdong/Shalang/0910	SL	Nephritis	12	GU938423
CK/CH/Guangdong/Shuitai/0901	ST1	Nephritis	27	GU938375
CK/CH/Guangdong/Shuitai/0903	ST3	Nephritis	12	GU938437
CK/CH/Guangdong/Wulian/0901	WL	Nephritis	20	GU938440
CK/CH/Guangdong/Xindadi/0902	XD2	Respiratory	9	GU938438
CK/CH/Guangdong/Xindadi/0903	XD3	Respiratory	27	GU938442
CK/CH/Guangdong/Xinnong/0811	XN	Nephritis	20	GU938365
CK/CH/Guangdong/Xinnong1/0901	XN1	Nephritis	16	GU938373
CK/CH/Guangdong/Xinnong2/0901	XN2	Nephritis	49	GU938374
CK/CH/Guangxi/Guilin1/0806	GL1	Nephritis	20	GU938390
CK/CH/Guangxi/Guilin2/0806	GL2	Nephritis	30	GU938396
CK/CH/Guangxi/Guilin/0811	GL	Respiratory	15	GU938389
CK/CH/Guangxi/Hezhou/0903	HEZ	Nephritis	47	GU938395
CK/CH/Guangxi/Luchuan/0906	LC	Nephritis	27	GU938394
CK/CH/Guangxi/Luchuan1/0910	LC1	Nephritis	15	GU938403
CK/CH/Guangxi/Luchuan2/0910	LC2	Nephritis	17	GU938404
CK/CH/Guangxi/Luchuan3/0910	LC3	Nephritis	12	GU938405
CK/CH/Guangxi/Luchuan4/0910	LC4	Nephritis	5	GU938417
CK/CH/Guangxi/Luchuan5/0910	LC5	Nephritis	11	GU938418
CK/CH/Guangxi/Luchuan6/0910	LC6	Nephritis	69	GU938419
CK/CH/Guangxi/Luchuan7/0910	LC7	Nephritis	15	GU938420
CK/CH/Guangxi/Luchuan8/0910	LC8	Nephritis	10	GU938422
CK/CH/Guangxi/Luchuan9/0910	LC9	Nephritis	65	GU938424
CK/CH/Guangxi/Nanning/0903	NN	Nephritis	33	GU938400
CK/CH/Guangxi/Yulin/0812	YL08	Nephritis	30	GU938391
CK/CH/Guangxi/Yulin/0904	YL4	Nephritis	25	GU938399
CK/CH/Guangxi/Yulin/0906	YL6	Nephritis	45	GU938393
CK/CH/Hainan/0811	HN08	Nephritis	35	GU938439
CK/CH/Hainan1/0901	HN1	Nephritis	17	GU938382
CK/CH/Hainan2/0901	HN2	Nephritis	15	GU938383
CK/CH/Hainan1/0903	HN31	Nephritis	20	GU938376
CK/CH/Hainan2/0903	HN32	Nephritis	30	GU938377
CK/CH/Hainan3/0903	HN33	Nephritis	25	GU938384
CK/CH/Hainan4/0903	HN34	Nephritis	21	GU938444
CK/CH/Hainan1/0904	HN41	Nephritis	46	GU938378
CK/CH/Hainan2/0904	HN42	Nephritis	17	GU938379
CK/CH/Hainan3/0904	HN43	Nephritis	44	GU938380
CK/CH/Hainan4/0904	HN44	Nephritis	48	GU938381
CK/CH/Hubei/Wuhan1/0901	WH1	Nephritis	18	GU938369
CK/CH/Hubei/Wuhan2/0901	WH2	Nephritis	18	GU938370
CK/CH/Hubei/Wuhan3/0901	WH3	Nephritis	24	GU938371
CK/CH/Hubei/Wuhan4/0901	WH4	Nephritis	20	GU938372
CK/CH/Jiangsu/Lianyungang/0902	LYG	Nephritis	28	GU938392
CK/CH/Jiangsu/NanJiao/0904	NJ	Respiratory	20	GU938412
CK/CH/Zhejiang/Huzhou1/0910	HZ1	Nephritis	64	GU938425
CK/CH/Zhejiang/Huzhou2/0910	HZ2	Nephritis	12	GU938426
CK/CH/Zhejiang/Huzhou3/0910	HZ3	Nephritis	32	GU938427
CK/CH/Zhejiang/Huzhou4/0910	HZ4	Nephritis	4	GU938428
CK/CH/Zhejiang/Huzhou5/0910	HZ5	Nephritis	12	GU938429
CK/CH/Zhejiang/Huzhou6/0910	HZ6	Nephritis	13	GU938430
CK/CH/Zhejiang/Huzhou7/0910	HZ7	Nephritis	15	GU938434
CK/CH/Zhejiang/Huzhou8/0910	HZ8	Nephritis	21	GU938435
CK/CH/Zhejiang/Huzhou9/0910	HZ9	Nephritis	16	GU938436
CK/CH/Zhejiang/Quzhou1/0910	QZ1	Respiratory	14	GU938431
CK/CH/Zhejiang/Quzhou2/0910	QZ2	Respiratory	12	GU938432
CK/CH/Zhejiang/Quzhou3/0910	QZ3	Respiratory	9	GU938433

After three passage propagation, IBVs of all isolates induced peripheric lesions and growth retardation of embryo at 72 h post-inoculation. Since the fourth day post-inoculation, most of the chicks were listless and huddled together, showed ruffled feathers. The results of virus recovery in chicks indicated 87.5% (70/80) isolates caused serious kidney lesions, which were presented with swollen specked kidney and distended ureters filled with uric acid were nephropathogenic type, and the other ten isolates in the study caused respiratory system signs, which were consistent with the clinical record of each strain (Table 1).

### Homologies among S1 nucleotide and deduced amino acid sequences

The obtained strains were characterized phylogenetically by nucleotide sequence analysis of the hyper-variable S1 gene of IBV. The nucleotide and amino acid sequence similarities between the eighty IB strains were ranging from 75.4% (strain CQ8 and HY) to 100% (strain PT1 and PT3) and 73.9% to 100%, respectively. Compared to the 28 reference strains published in the GenBank, the identity of the nucleotide and amino acid sequence among the 108 isolates (including the 80 isolates in this study plus the 28 reference strains) were 75.1 to 99.8% and 73.1 to 99.8%, respectively, indicating low homology and high variation among the isolated and reference strains.

### Mutation analysis

As shown in the Table 2 and Table 3, S1 genes of the newly strains contain mutations, insertions and deletions, resulting in different lengths of nucleotides. S1 genes of these strains were generated and confirmed from three time sequencing results, contained 1641, 1647, 1650, 1653, 1656, 1659 and 1662 nucleotides, amino acids sequences ranging from 547 (LC strain) to 554 (LC strain). The length differences indicated amino acid insertions and deletions exist among the different strains.

**Table 2:** Sequence alignment of amino acid residues of the S1 glycoprotein of IBV strains with the M41 strain

Strains	**Deletions**^**1**^	**Insertions**^**2**^	Cleavage recognition motifs
CQ8		119(2)GL, 140(2)NS	RRTGR^a^
CQ9	21(1)D, 118(1)G	72(7)YTNGNDV	HRRRR^b^
MS1	21(1)D, 118(1)G	72(7)YTNGNDV	HRRRR^b^
PT1	21(1)D, 118(1)G	72(7)YTNGNDV	RRSRR^c^
PT2		117(2)GV, 286(1)N	RRLRR^d^
PT3	21(1)D, 118(1)G	72(7)YTNGNDV	RRSRR^c^
PT4	21(1)D, 118(1)G	72(7)YTNGNDV	RRSRR^c^
BT		119(2)GS	RRFRR^e^
Y4		117(2)GI, 286(1)N	RRFRR^e^
Y9		117(2)GI, 286(1)N	RRFRR^e^
FML	21(1)D, 118(1)G	72(7)YTNGNDV	RRFRR^e^
HY		117(2)GV, 286(1)N	RRLRR^d^
HY41	21(1)D, 118(1)G	72(7)YTNGNDV	RRFRR^e^
HY42	21(1)D, 118(1)G	72(7)YTNGNDV	RRFRR^e^
HY51	23(1)S, 118(1)G	72(7)YTNGNDV, 286(1)G	RRFRR^e^
HY52	23(1)S, 118(1)G	72(7)YSNGNDV	RRSKR^f^
HY1	21(1)D, 118(1)G	72(7)YTNGNDV	RRFRR^e^
HY2	23(1)S, 118(1)G	72(7)YSNGNDV	RRSKR^f^
HY3	21(1)D, 118(1)G	72(7)YTNGNDV	RRFRR^e^
KY		24(1)N, 119(2)GS	HRRRR^b^
LZ1		24(1)N, 119(2)GS	HRRRR^b^
LZ2		24(1)N, 119(2)GS	HRRRR^b^
LZ3		24(1)N, 119(2)GS	HRRRR^b^
LH	21(1)D, 118(1)G	72(7)YTNGNDV	RRFRR^e^
SL		24(1)N, 119(2)GS	HRRRR^b^
ST1		119(2)GS	RRFRR^e^
ST3		119(2)GS	RRFRR^e^
WL	21(1)D, 118(1)G	72(7)YTNGNDV	HRRRR^b^
XD2		119(2)GS	RRFRR^e^
XD3		24(1)N, 119(2)GS	RRFRR^e^
XN		24(1)N, 119(2)GS	RRFRR^e^
XN1		25(1)N, 119(2)GS	HRRRR^b^
XN2		119(2)GS	RRFRR^e^
GL1	21(1)D, 118(1)G	72(7)YTNGNDV	HRRRR^b^
GL2		117(2)GI, 289(1)N	RRLRR^d^
GL		25(1)N, 119(2)GS	HRRRR^b^
HEZ	21(1)D, 118(1)G	72(7)YTNGNDV	RRFRR^e^
LC	118(1)G	72(7)YTNGNDV, 286(1)S	RRFRR^e^
LC1		117(2)GI	RRFRR^e^
LC2		117(2)GI	RRFRR^e^
LC3		117(2)GI	RRFRR^e^
LC4		117(2)GI	RRFRR^e^
LC5		117(2)GI	RRFRR^e^
LC6	21(1)D, 118(1)G	72(7)YTNGNDV	RRFRR^e^
LC7		117(2)GI	RRFRR^e^
LC8		117(2)GI	RRFRR^e^
LC9	21(1)D, 118(1)G	72(7)YTNGNDV	RRFRR^e^
NN		117(2)GI, 286(1)N	RRLRR^d^
YL08		117(2)GI, 286(1)N	RRLRR^d^
YL4	24(1)S, 118(1)G	72(7)YTNGNDV	RRFRR^e^
YL6		24(1)N, 119(2)GS	HRRRR^b^
HN08		119(2)GS	RRFRR^e^
HN1	21(1)D, 118(1)G	72(7)YTNGNDV	RRFRR^e^
HN2	21(1)D, 118(1)G	72(7)YTNGNDV	RRFRR^e^
HN31	21(1)D, 118(1)G	72(7)YTNGNDV	RRFRR^e^
HN32	21(1)D, 118(1)G	72(7)YTNGNDV	RRFRR^e^
HN33	21(1)D, 118(1)G	72(7)YTNGNDV	RRFRR^e^
HN34	21(1)D, 118(1)G	72(7)YTNGNDV	RRFRR^e^
HN41	21(1)D, 118(1)G	72(7)YTNGNDV	RRFRR^e^
HN42	21(1)D, 118(1)G	72(7)YTNGNDV	RRFRR^e^
HN43	21(1)D, 118(1)G	72(7)YTNGNDV	RRFRR^e^
HN44	21(1)D, 118(1)G	72(7)YTNGNDV	RRFRR^e^
WH1		24(1)N, 119(2)GS	HRRRR^b^
WH2		24(1)N, 119(2)GS	HRRRR^b^
WH3		25(1)N, 119(2)GS	HRRRR^b^
WH4		25(1)N, 119(2)GS	HRRRR^b^
LYG		25(1)N, 119(2)GS	HRRRR^b^
NJ			RRFRR^e^
HZ1		25(1)N, 119(2)GS	HRRRR^b^
HZ2		25(1)N, 119(2)GS	HRRRR^b^
HZ3		25(1)N, 119(2)GS	HRRRR^b^
HZ4	21(1)D, 118(1)G	72(7)YTNGNDV	RRFRR^e^
HZ5		25(1)N, 119(2)GS	HRRRR^b^
HZ6		25(1)N, 119(2)GS	HRRRR^b^
HZ7		25(1)N, 119(2)GS	HRRRR^b^
HZ8		25(1)N, 119(2)GS	HRRRR^b^
HZ9		25(1)N, 119(2)GS	HRRRR^b^
QZ1		24(1)N, 119(2)GS	HRRRR^b^
QZ2		24(1)N, 119(2)GS	HRRRR^b^
QZ3		24(1)N, 119(2)GS	HRRRR^b^

^1 ^positions of residues in deduced amino acid sequences of the S1 protein of the M41 vaccine strain;

^2 ^positions of residues in deduced amino acid sequences of the S1 protein of the M41 strain between which the residue(s) of other IBVs was (were) inserted.

a, RRTGR: Arg-Arg-Thr-Gly-Arg (1/80); b, HRRRR: His-Arg-Arg-Arg-Arg (28/80); c, RRSRR: Arg-Arg-Ser-Arg-Arg (3/80); d, RRLRR: Arg-Arg-Leu-Arg-Arg 5/80; e, RRFRR: Arg-Arg-Phe-Arg-Arg (41/80); f, RRSKR Arg-Arg-Ser-Lys-Arg (2/80).

**Table 3:** Different lengths of nucleotides and deduced amino acids of S1 glycoprotein gene of the 80 isolated IBV strains

Length (nt/aa)	Strains
1641/547	NJ
1647/549	BT, ST1, ST3, XD2, XN2, LC1, LC2, LC3, LC4, LC5, LC7, LC8, HN08
1650/550	PT2, Y4, Y9, HY, KYJD, LZ1, LZ2, LZ3, SL, XD3, XN, XN1, GL2, GL, NN, YL08, YL6, WH1, WH2, WH3, WH4, LYG, HZ1, HZ2, HZ3, HZ5, HZ6, HZ7, HZ8, HZ9, QZ1, QZ2, QZ3
1653/551	CQ8
1656/552	CQ9, MS1, PT1, PT3, PT4, FML, HY41, HY42, HY52, HY1, HY2, HY3, LH, WL, GL, HEZ, LC6, LC9, HN1, HN2, HN31, HN32, HN33, HN34, HN41, HN42, HN43, HN44, HZ4
1659/553	HY51, YL4
1662/554	LC

Through the alignment analysis, the deletions, insertions and mutations of each obtained S1 gene was summarized in Table 2. Most variations in the deduced amino acid sequences of Chinese IBVs were observed among residues 63-69, 211-212 and 354-358 (numbering was with reference to S1 sequence of the Mass41 strain).

The precursor protein of S glycoprotein is cleaved into amino-terminal S1 and S2 protein by the protease during viral maturation [9]. In this study, the most common cleavage recognition sites of S1 gene were RRF(S/L) RR (49/80) or HRRRR (28/80) in the China field strains (Table 3). The exceptional ones included CQ8 (RRTGR), HY52 (RRSKR), and HY2 (RRSKR). The cleavage sites of these two strains containing amino acids K, T, and G, were novel motifs compared to the reference strains, and quite different with the other isolates of the cleavage site.

### Phylogenetic analysis of the isolated strains

**Figure 1:**
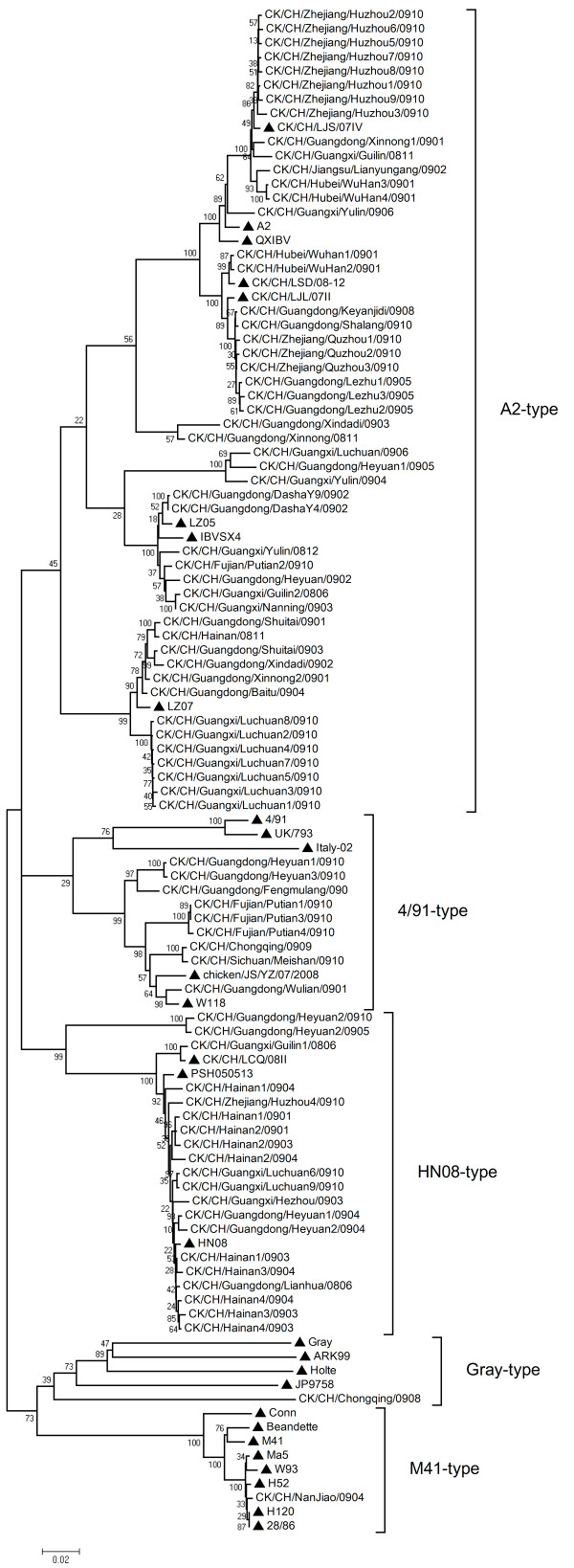
**The phylogenetic tree of IBVs isolated in mainland China during 2008-2009 based on the viral S1 sequences**. The reference strains in this study were marked with "▲".

A phylogenetic tree was constructed from the nucleotides sequences of the S1 glycoprotein genes. As shown in the Figure 1, the 80 isolates IBV strains were clustered into five distinct genetic groups or genotypes which were considerably heterogeneous, including A2-type (49 newly isolated strains), 4/91-type (9 newly isolated strains), HN08-type (20 newly isolated strains), Gray-type and M41-type. The newly isolated strains mainly belonged to A2-type, 4/91-type and HN08-type branch. The phylogenetic relationship of strains at different times and geographical regions displayed complexity and diversity.

Strains isolated from Hubei, Zhejiang, Jiangsu, Guangdong, Guangxi and Fujian province mainly belonged to the A2 branch, also including other seven published IBV strains from China (QXIBV, CK/CH/LJL/07II, CK/CH/LJS/07IV, CK/CH/LSD/08-12, IBVSX4, LZ05 and LZ07). The isolated strains of Hainan province and a few isolated strains from Guangdong and Fujian province belonged to the HN08 branch, included PSH050513 and CK/CH/LCQ/08II. Group Gray-type was correlative with the American strain (Gray), included other two classical American strains (ARK99 and Holte), one Japanese strain (JP9758), and the exceptional field strain (CQ08). Most of the current vaccine strains (H120, H52, Ma5, M41, W93, 4/91 and 28/86) were belonged to the M41 branch, which including one field strains (NJ). However, the current pandemic strains were mostly 4/91-type, A2-type (QXIBV-type) and HN08-type, indicating that the field IBVs co-circulating in chicken flocks in China were evolutionarily distant from the known vaccine strains.

## Discussion

Infectious bronchitis (IB) is one of the most common and difficult-control poultry diseases in China, caused persistent but infrequent outbreaks in commercial chicken farms [24,25,28]. Commercial vaccines based on H120, H52, 28/86, Ma5, W93 and M41 strains, have been widely used to control the disease [2,29]. Natural outbreaks of IBV often are the result of infections with strains that differ serologically from the vaccine strains. Come to the rapid and complicated evolutionary of IBV, it is imperative to learn profoundly the circulating IBVs, facilitate selecting the candidate vaccine strain against the infections [2,24].

In this study, 80 IBV strains were isolated from the vaccinated chicken flocks, with a wide age range of IB outbreak. The chickens infected before the age of 5 days which might be caused by the vertical transmission of IBVs or the maternal antibody could not provide pertinent protection against the prevalent strains [30]. Furthermore, there was accumulating evidence indicated that the nephropathogenic IBVs have become prevalent in China in last several years [23,26,31]. Through clinical records and the virus recovery trials, 70 identified isolates mainly caused typical swollen kidney, different from the respiratory type strains isolated in earlier years, including the major vaccine strains. These findings indicated that all 80 isolated IBV strains from China during 2008-2009 were evolutionarily distant from the vaccine strains used for current, resulting in vaccination failure cases.

The S1 protein determined the serotypic evolution, the phenotype change and the genetic diversity of IBVs [32]. In the present study, nucleotide and derived amino acid sequences of S1 protein genes of the 80 field strains were aligned and compared to the representative strains, to determine the relationship of circulating field isolates, vaccine strains and previously described variant strains. Newly isolated strains shared between 75.4% to 100% nucleotide sequence similarity with each other, higher similarity than the vaccine strains and other representative IBVs. Although the IBVs all over the world shared some common antigenic types, virus strains within a geographic region were unique and distinct, even in different provinces of China. The variants were mostly located in the first 300 amino acids in the N-terminal of the S1 protein of IBV, even though the mutants consisted of insertions, deletions and point mutations were complicated and detailedly different, the hypervariable regions in S1 protein in this study were similar to previous studies [19,23,26].

The phylogenetic analysis showed that there were five subgroups of IBVs co-circulating in China, and multiple strains might cause the constant IB outbreaks. The newly isolated strains were mostly derived from A2, 4/91 and HN08. Only CK/CH/Chongqing/0908 belonged to the branch of Gray. The phylogenetic distributions were closely relative to geographical factors. Most of the recently isolated IBVs in this study formed the distinct cluster related to the A2 type. However, the routine vaccine strains mainly belong to M41-type branch. A2 strain is closely related to 4/91 serotype, spreading over Europe since its first isolation in UK in 1991 [9,24,33-37]. In this study, 61.3% (49/80) field isolates belonged to the A2-type branch, which included 85.7% (42/49) nephropathogenic field isolates of this study. The QXIBV, first isolated in China and reported associated predominantly with various forms of renal pathology in China, was also representative A2-type strain [25,31]. The analysis results were according to the prevalence of nephropathogenicity IB. To date, the QX-like IBV strains have been widely isolated in many European countries, and become a dominant genotype [5,38]. Through IB surveys, the European QX-like IBV strains have been reported that caused 86% respiratory signs, 22% litter or enteric problems, only 2% had swollen kidneys [39]. Absorbingly, the QX-like IBV strains have undergone divergent evolution paths, brought out different variants in Europe and China. Similarly, seven exceptional strains located in the A2-type branch caused evident respiratory problems, including three isolates from Zhejiang province (QZ1, QZ2 and QZ3) and three isolates from Guangdong province (XD2, XD3 and LZ2), and GL from Guangxi province. The results of our study indicated the strain grouping, such as phenotype and genotype, were not only depended on the geographical factors. The evolutionary pace and the epidemiology characteristics of the IBV were complicated.

## Conclusions

In conclusion, the data obtained from our study suggest most of present IBV isolates in China are A2-like nephropathogenic strains. To control the prevalence and well prepare for the potential outbreaks of IB, the candidate virus strain for vaccination might be selected timely and specifically in a geographical region, which manifests the importance of continuing surveillance of new IBV strains. This paper is a periodic report on our ongoing surveillance program. We hope the study could contribute to guiding the development of effective vaccines and establishment of control policy for IB.

## Materials and methods

### Viruses

During the period from June 2008 to November 2009, circulating field IBV isolates were selected from suspected broilers and broiler breeders in vaccinated flocks from eastern, southern, southwestern and central China. Documented clinical signs of the birds included typical respiratory and nephropathogenic IB symptoms and pathological changes. The homogenized tissue pool of kidney and trachea of the field isolates collected from the chickens infected naturally were frozen and thawed three times, treated with 200 μg/ml gentamicin and 200 U/ml penicillin and centrifuged at 7,000 × g. After incubating for three hours at 4°C, the supernatant samples were propagated by inoculating via the allantoic cavity with 0.2 ml of each isolate into three 10 day old SPF embryonated eggs for more than three passages. All the isolated strains were verified by the observation of curled and dwarfed embryos. The embryos dying within 24 hours of inoculation were discounted and screened to be nonspecific deaths.

The viruses were further confirmed by RT-PCR assay. Total RNA extraction of the allantoic fluid was completed using RNAiso reagent (TaKaRa Biotechnology, Dalian, China) according to the manufacturers' instructions. Reverse transcription polymerase chain reaction (RT-PCR) was carried out by PrimeScriptTM One-Step RT-PCR Kit with the IBV primers (National standard, GBT23197-2008), one primer pair targeting the M gene (Ms: 5'-CCTAAGAACGGTTGGAAT-3', Mx: 5'-TACTCTCTACAC ACACAC-3') and another pair for the 3' UTR of genome (3's: 5'-GGAAGATAGGCATGTAGCTT-3', 3'x: 5'-CTAACTCTATAC TAGCCTAT-3').

The allantoic fluids containing IBV isolates after 72 h post inoculation were harvested for subsequent experiments, and the remains were preserved in liquid nitrogen.

### Virus recovery

Five 1-day-old SPF White Leghorn chickens were intranasally inoculated with filtration sterilized allantoic fluid of each isolated virus strains, respectively. All of the chicks were examined and recorded daily for clinical signs of infection and mortality for 20 days post-inoculation, the dead birds were necrospied for lesions of respiratory tract or nephritis. Finally, all the survivors were sacrificed and necrospied.

### RT-PCR and S1 gene sequencing

A pair of specific primers was designed to amplify the entire S1 protein gene, including the forward primer (S1F): 5'-AAGACTGAACAAAAGACCGACT-3', and the reverse primer (S1R): 5'-CAAAACCTGCCATAACTAACATA-3'. Reverse transcription and amplification were performed using the PrimeScriptTM One-Step RT-PCR Kit in 25 μl reaction volume containing 20 μl of RT-PCR PreMix (reaction buffer, dNTPs, 2 μl of enzyme mix), 2 μl of extracted viral RNA and the specific primer pair. Reverse transcription and amplification were performed as one cycle of 50°C for 30 min, 94°C for 2 min, followed by 30 cycles of denaturation at 94°C for 40 s, annealing at 51°C for 40 s and extension at 72°C for 2 min, respectively) with a final 10 min extension step at 72°C. The PCR products were cloned into pMD19-T vector (TaKaRa Biotechnology, Dalian, China) for later sequencing (AuGCT Biotechnology, Beijing, China).

### Genetic variability and phylogenetic analysis

The S1 protein gene sequences obtained in this study were submitted to the GenBank database and assigned the accession numbers of GU471864-GU471897, GU471793-GU471805 (Table 1).

Twenty-eight representative sequences available in GenBank were contributed to comparison and phylogenetic analysis in this study, including vaccine strains, H120 (Accession numbers: M21970), H52 (AF352315), Ma5 (AY561713), W93 (AY427818), 4/91 (AF093793), 28/86 (AY846750), M41 (DQ834384); well-known non-Chinese strains for subgrouping, A2 (AY043312), ARK99 (M99482), Beandette (X02342), Conn (L18990), Gray (L14069), Holte (L18988), Italy-02 (AJ457137), JP9758 (AY296746), UK/7/93 (Z83979); and the representative strains isolates from China, chicken/JS/YZ07/2008 (FJ807653), CK/CH/LCQ/08II (GQ258305), CK/CH/LJL/07II (FJ345374), CK/CH/LJS/07IV (FJ345378), CK/CH/LSD/08-12 (GQ258327), HN08 (GQ265940), IBVSX4 (FJ793939), LZ05 (GQ265943), LZ07 (GQ265944), PSH050513 (DQ160004), QXIBV (AF193423), W118 (DQ679420). The multiple-alignment was carried out using DNAStar sequence analysis software (DNAStar *Inc.*, Madison, WI, USA). The phylogenetic tree was constructed using the MEGA 4.1 software with neighbor-joining method and each tree was produced using a consensus of 1000 bootstrap replicates [40].

## Competing interests

The authors declare that they have no competing interests.

## Authors' contributions

JJ and JX carried out most of the experiments and wrote the manuscript, and should be considered as first authors. FC and QX critically revised the manuscript and the experiment design. DS, KZ, CX, JQ, HL, JM and YB helped with the experiment. All of the authors read and approved the final version of the manuscript.
